# Modelling of microstructural evolution in multi-layered overlay coatings

**DOI:** 10.1007/s10853-017-1365-2

**Published:** 2017-07-17

**Authors:** M. S. A. Karunaratne, M. A. E. Jepson, N. J. Simms, J. R. Nicholls, R. C. Thomson

**Affiliations:** 1 Department of Materials, Loughborough University, Loughborough, Leicestershire LE11 3TU UK; 2 Power Engineering Centre, Cranfield University, Cranfield, Bedfordshire MK43 0AL UK

## Abstract

Functionally graded, multi-layered coatings are designed to provide corrosion protection over a range of operating conditions typically found in industrial gas turbines. A model incorporating diffusion, equilibrium thermodynamics and oxidation has been developed to simulate the microstructural evolution within a multi-layered coating system. The phase and concentration profiles predicted by the model have been compared with an experimental multi-layered system containing an Al-rich outer layer, a Cr-enriched middle layer and an MCrAlY-type inner layer deposited on a superalloy substrate. The concentration distribution and many microstructural features observed experimentally can be predicted by the model. The model is expected to be useful for assessing the microstructural evolution of multilayer coated systems which can be potentially used on industrial gas turbine aerofoils.

## Introduction

To enable progressively higher firing temperatures and pressures in industrial gas turbines, the alloys used in their hot gas paths have needed considerable development since the 1960s. Initially, these developments coupled increased resistance to creep and fatigue with better oxidation and hot corrosion resistance. However, since the 1970s, base alloys have been developed with increasingly optimised mechanical load capabilities, but at the cost of reduced corrosion resistance, through lowering Cr content and by addition of Al, Ti and refractory metals (Al and Ti support the formation of γ′, while refractory metal additions provide solid solution strengthening to the γ phase). Thus, coatings that protect the base alloys from the surrounding environment have become critical parts of hot gas path components. Coatings have been developed to provide either: (a) a hot corrosion/oxidation-resistant barrier, and/or (b) a low conductivity thermal barrier coating (TBC) to reduce cooling air requirements and/or base alloy operating temperatures. With the increasingly large gas–metal temperature difference, the roles of coating systems have become more important.

**Figure 1:**
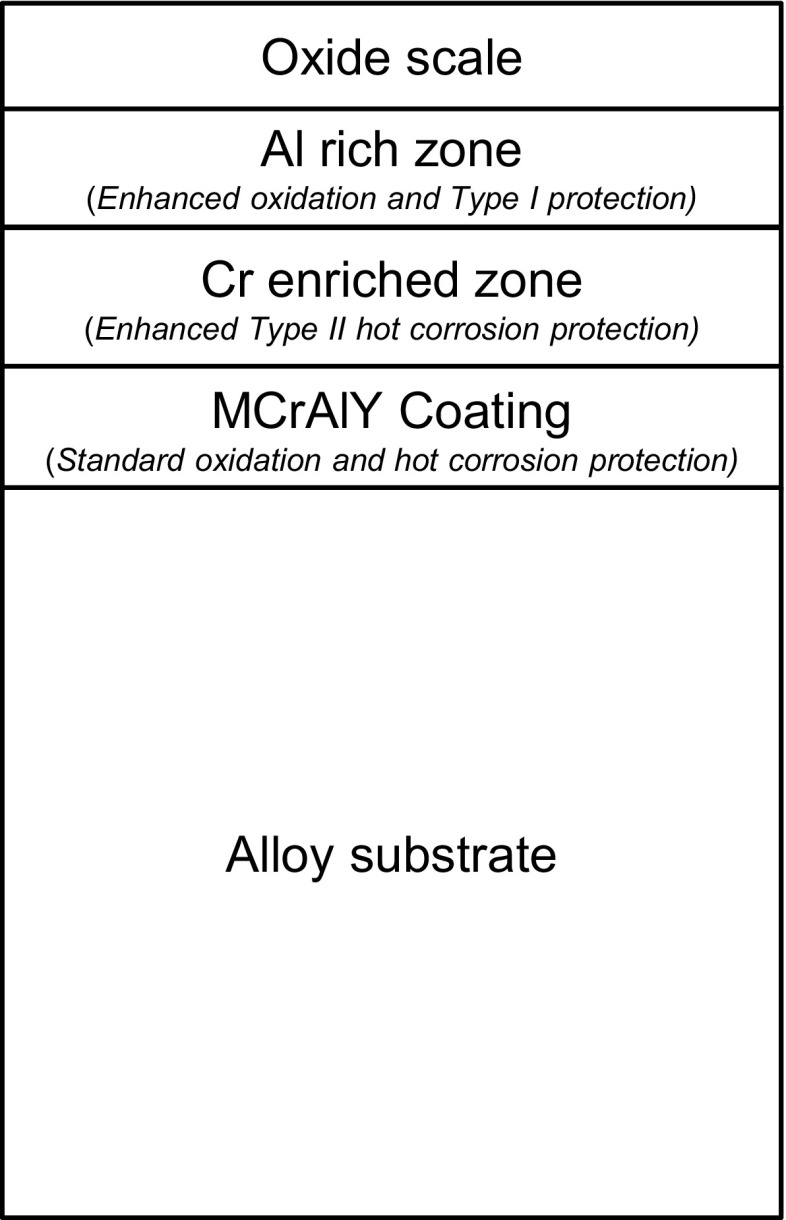
Illustration of a typical multilayer coating configuration (not to scale)

Different types of coatings have been developed to meet the needs of particular types of gas turbine environments, and these have been reviewed elsewhere, e.g. [1, 2]. However, the design of current gas turbine components is such that a wide range of conditions can be experienced by a single component. Without a TBC, variations in gas and metal temperatures, local gas compositions and flow regimes can result in conditions that cause oxidation, Type I and Type II hot corrosion at different locations on a surface of a single component. Thus, coating systems are needed that can resist the different degradation routes. This has provided the driving force for the development of ‘SMART’ coatings that are able to resist multiple degradation routes. One route to develop such coatings is using the functionally graded materials concept where multi-layered coatings are generated on a component surface [3–5]. In this approach, each layer has a specific role to play in providing the coating with its desired properties (e.g. Fig. 1). However, a particular concern for such complex coating systems is their thermal stability; during operation at high temperatures, the distinct coating layers need to be maintained for long periods and not degrade via interdiffusion or surface reactions.

Computer modelling has been successfully applied in many areas of nickel-based superalloys such as thermodynamics [6–8], solidification [9, 10], microstructural evolution [11, 12] and prediction of physical properties [10, 13]. A modelling capability that can predict microstructural evolution processes in coating structures is extremely valuable and can assist the development process of advanced coating systems such as SMART coatings. The development of a microstructural simulation model for coated superalloy systems was reported earlier [12], and the model was validated against a number of experimental single-layered coating systems [12, 13]. In this work, the capability of the model has been extended further to incorporate multi-layered coatings and the developed model is used to examine the thermal stability of a trial, functionally graded, multi-layered coating system from an early stage of a coating development process.

## Experimental procedure

An example of a chemically graded coating structure was used for this study. An argon-shrouded plasma spray process was used to deposit a NiCrAlY base coating on a superalloy substrate. This was followed by a second step aimed at surface treating the as-sprayed NiCrAlY to form a chromium-rich interlayer (this step is proprietary, but is designed to produce a continuous inner zone containing some 60 wt% Cr). Then, an aluminising treatment was carried out to develop an outer β-NiAl [3, 4]. Details of heat treatments carried out during the processing of this multi-layered coating system are also proprietary.

The coated samples [3] presented within this article are used for model verification purposes only and belong to an early generation of multilayer coatings. Work is ongoing to improve these coatings for use within relevant industrial applications. The aim of this research is to demonstrate the applicability and validity of a model developed for simulating multi-layered coating systems which can potentially be used for gas turbine components.

The samples had been subjected to a proprietary high-temperature coating treatment anneal followed by ageing in air −300 vpm SOx either at 700 °C (Sample 1) or 900 °C (Sample 2) for 500 h. The aged samples were mounted in a low-temperature curing, edge-retaining resin, ensuring that the coating deposition direction was parallel to the surface of the mount. The mounted samples were ground and polished using oil-based preparation procedures using successively finer silicon carbide paper and finally 9-, 3- and 1-µm diamond polish. To ensure that the specimens were suitable for electron microscopy characterisation, they were coated with a thin layer of gold. This layer was applied using a Quorum Technologies Emitech SC7640 sputter coater with a coating time of approximately 20 s.

Scanning electron microscopy was carried out using a Leo 1530VP field emission gun scanning electron microscope (FEGSEM) operating in backscattered electron (BSE) mode with an accelerating voltage of 20 kV and a working distance of approximately 10 mm. The detector used was a 4-quadrant detector operating with all quadrants in their normal operation mode. For energy-dispersive X-ray spectroscopy (EDS), an EDAX TEAM Pegasus system was used and the concentration profiles of the coating were obtained by using the multipoint (matrix) function where a matrix of 10 rows of 50 analysis areas was collected and each column of the matrix was averaged to give a smooth curve suitable for comparison to the model output.

Preparation of transmission electron microscopy samples was carried out using focused ion beam (FIB) milling which allowed site-specific transmission electron microscope (TEM) sample preparation. These samples, measuring approximately 25 µm × 5 µm, were produced using an FEI Nova Nanolab 600 operating at an accelerating voltage of 30 kV throughout, with the assistance of an Omniprobe micromanipulator for lamella extraction. Lamellae were attached to copper half-grids using platinum deposition and polished using a final beam current of 300 pA to a thickness of less than 200 nm.

Examination of TEM specimens was carried out using a JEOL 2000FX microscope operating at an accelerating voltage of 200 kV. Images were collected using a Gatan Erlangshen charge-coupled digital camera, and EDS data were collected using an Oxford Instruments Inca system.

## Simulation model

### Diffusion model

Due to the planar nature of interfaces between coating layers and also between coating layers and the substrate, diffusion occurring in a typical multilayer coating system could be approximated by a one-dimensional model. Hence, the diffusion of elements within coating layers and substrate was modelled using the 1-D multicomponent representation of Fick–Onsager law [14, 15] given by Eq. (1)

1 $$ \frac{{\partial C_{i} }}{\partial t} = \mathop \sum \limits_{j = 1}^{n - 1} \left\{ {\tilde{D}_{ij}^{n} \frac{{\partial^{2} C_{j} }}{{\partial x^{2} }} + \left( {\mathop \sum \limits_{k = 1}^{n - 1} \frac{\partial }{{\partial C_{k} }}\tilde{D}_{ik}^{n} \frac{{\partial C_{k} }}{\partial x}} \right)\frac{{\partial C_{j} }}{\partial x}} \right\} $$

where *i*, *j* and *k* are chemical elements. The interdiffusion coefficient matrix $\tilde{D}_{ij}^{n}$ is expressed in relation to a solvent *n*, which is Ni for the present system, and *C*_*i*_ represents the concentration of element *i*.

### Diffusion coefficients

Interdiffusion coefficients in Ni-FCC phase were obtained from the indicated references for elements: Co [16], Mo [17], Ti [18], Re [19], W [19], Ta [19], Al [20] and Cr [21]. For the γ′ phase, diffusion coefficients reported in [22] for Al, [23] for Ti, and [24] for Co and Cr, were incorporated. For the β phase diffusion coefficients of Al provided in [25] were used. Since diffusion data were unavailable for elements within TCP phases and, and since diffusivity was expected to be slow in ordered TCP phases and BCC α-Cr phase, their diffusion coefficients were assumed to be a fraction (5%) of that in the FCC-Ni phase. The concentration dependences of diffusion coefficients were modelled by fitting third-degree polynomials to published data, and their temperature dependences were modelled by assuming Arrhenius behaviour. Full details of the modelling process and fitted parameters are given in [11] and hence not repeated here. For each node, an effective diffusion coefficient for each element was calculated by taking a volume weighted average of concentration-dependent diffusion coefficients in each phase.

### Oxidation model

For oxidation, the model proposed by Meier et al. for a Ni–Co–Cr-based bond coat was used [26]. The model assumes that only Al is oxidised at the coating surface and the diffusion of elements within the oxide was not considered in the current model. For isothermal oxidation, the boundary condition at the oxide/coating interface is given in [26] as the rate of Al consumption, where the thickness *δ* of the oxide scale in is given by Eq. (2)

2 $$ \delta = \left[ {\exp \left\{ {Q\left( {\frac{1}{{T_{0} }} - \frac{1}{T}} \right)} \right\}t} \right]^{n} $$

where *Q* is a constant and equal to 27777.4, *T* is the temperature in Kelvin, *T*_0_ is 2423.7 K, *t* is time in seconds and *n* is equal to 0.332. The scale thickness predicted by the simulation was validated against those given by (2), and these values were found to be in excellent agreement with each other across the time-step values used in current simulations.

### Thermodynamic model

The thermodynamic equilibrium calculations were performed using the application interface [27] of MTDATA [6]. The MTDATA program consists of a numerical technique for the minimisation of Gibbs free energy of a chemical system and was used in conjunction with a thermodynamic database for Ni-based superalloys, Ni-DATA [7, 8]. The code calls the MTDATA application interface at each time step, with the concentration at each node in the diffusion grid sequentially. The thermodynamic calculations, in turn, return a description of equilibrium phases which are likely to be present at each node. This description includes the fractional phase constitution and the composition within each of the phases.

Each thermodynamic calculation is computationally expensive because of the inclusion of a large number of elements and alloy phases. Furthermore, there is a requirement to perform a large number of such calculations due to the need to solve at each spatial grid point at every time step. Therefore, to address these problems, the simulations were performed in parallel so as to minimise the computational run time. This was achieved by partitioning the spatial grid points among multiple processors, which was possible as the thermodynamic calculations at each spatial grid point were independent.

### Grid Scheme

In the model, the differential terms in (1) were replaced by their finite-difference (F-D) equivalents as detailed in [26]. The explicit scheme was used to solve for concentrations of all elements. At the start, the F-D grid zones were located only in areas where concentration gradients were expected to be steep. It allowed computational resources to be concentrated more efficiently only where chemistry was changing, i.e. (a) near the outer oxidation layer, (b) between coating layers and (c) near the coating layer/substrate boundary. In all cases, interdiffusion zones were created. Initially, regions away from these zones had zero concentration gradients, and hence no concentration changes occurred as interdiffusion fluxes were absent. The presence of semi-infinite boundary conditions at termini of each grid zone was assumed, except near the oxidation/coating interface where the boundary condition was provided by the scale formation process instead.

The F-D grid zones were expanded dynamically into the coating layers (or substrate) as the concentration fields extended with time [26]. The interface between the outer coating layer and the oxide scale was treated as a moving phase boundary, using the scheme suggested in [28]. The expansion of zones continued until any two neighbouring zones overlapped (soft impingement) and from that stage, the diffusion zones were merged together to form a single zone.

### Simulation conditions

The multilayer coating system consisted of three layers on a superalloy substrate. The outermost and central layers were Al- and Cr-enriched, respectively, and the innermost layer was a NiCrAlY. The samples had been subjected to a proprietary high-temperature coating treatment anneal followed by ageing in air −300 vpm SOx either at 700 °C (Sample 1) or 900 °C (Sample 2) for 500 h. These thermal treatments were simulated using the model. The two samples had received different treatment conditions when the outer layers were deposited [4], hence resulting in compositional differences in that layer as illustrated in Table 1. The substrate composition was determined to be Ni–7.4Al–9Co–6Cr–0.6Mo–6.5Ta–1.0Ti–3.0Re–6.0 W (wt%) using EDS, and this composition was used in the simulations.

**Table 1:** Composition and thickness values of layers used in simulations

Layer	Composition (wt%)			Thickness (μm)
	Al	Cr	Ni	
700 °C				
1	28	45	27	44
2	9	50	41	41
3	10	20	70	200
900 °C				
1	45	15	40	50
2	8	54	38	40
3	10	22	68	210

Layer 1 is the outermost and layer 3 is the innermost

The initial grid layout consisted of four equispaced grid zones, each having 36 grid points; from outer to inner zone the grid spacings were 0.13, 0.24, 0.79 and 1.14 at 700 °C and 0.14, 0.26, 0.71 and 1.2 at 900 °C. The time step for the simulations was varied in accordance with stability criteria for the explicit finite-difference solution scheme, e.g. [26]. The density of the outer coating layer and partial molar volume of Al in the coating were assumed to be 7754 kg/m^3^ and 7.1 × 10^−6^ m^3^/mol. An initial scale thickness of 0.3 µm was allowed to be present in the samples. Other conditions used with the model can be found in [11].

## Results and discussion

### Microstructure of aged multilayer coatings

#### Coating microstructure

**Figure 2:**
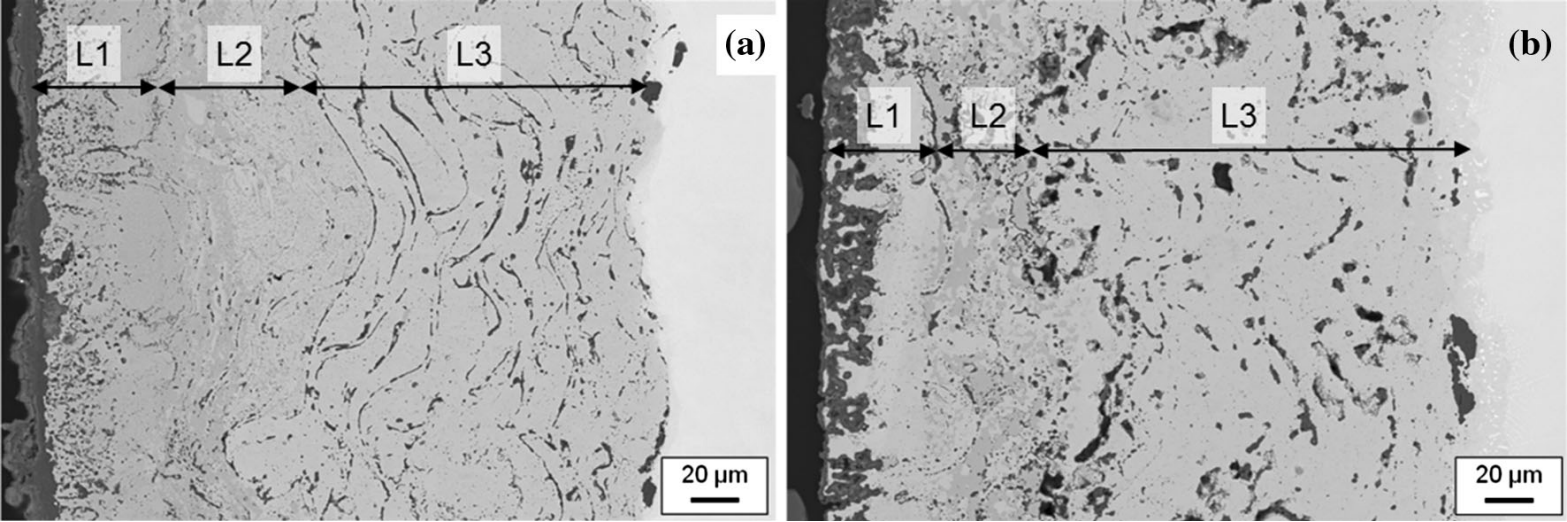
Backscattered electron micrographs of **a** Sample 1 and **b** Sample 2 aged for 500 h at 700 and 900 °C, respectively. *L1*, *L2* and *L3* identify the layers 1–3

The backscattered electron (BSE) images of the coating structure in Fig. 2 reveal the presence of some porosity along with significant variability of the layer thicknesses. This is a consequence of the coatings being part of the first generation of multilayer structures within a development programme. These first-generation coatings nevertheless are useful for model validation for chemical and phase distribution predictions in multilayer coatings.

The coating consists of three layers which are clearly discernible from the BSE images in Fig. 2 as differences in structure and contrast. In particular, the Cr-rich zone in Sample 2 has areas of lower image intensity relative to the surrounding matrix.

**Figure 3:**
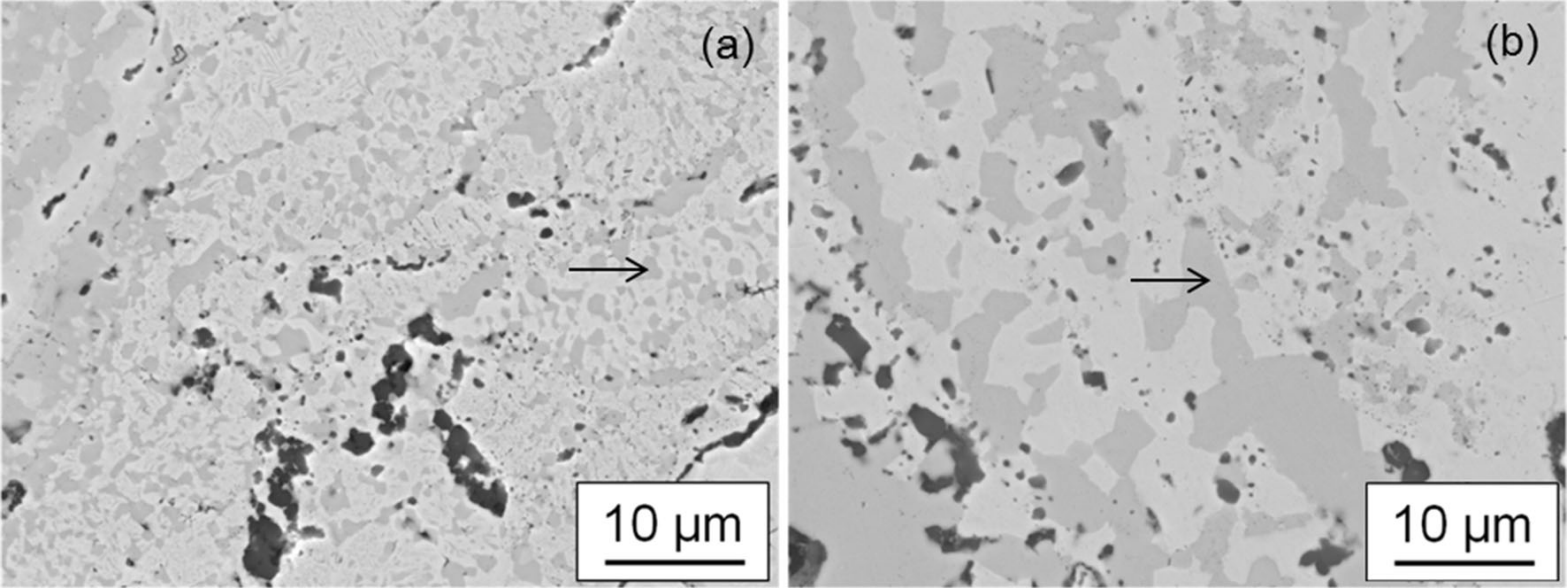
Backscattered electron micrographs of the Cr-rich zone from **a** Sample 1 and **b** Sample 2

The higher magnification BSE images in Fig. 3 are taken from the Cr-rich zone in Sample 1, Fig. 3a and Sample 2, Fig. 3b. It can be seen that in Sample 1 the dark regions (marked with an arrow) measure approximately 1 µm whereas in Sample 2 the darker phase is much larger with sizes exceeding 10 µm. Identification of this phase will be discussed in a later section.

#### Chemical distributions

**Figure 4:**
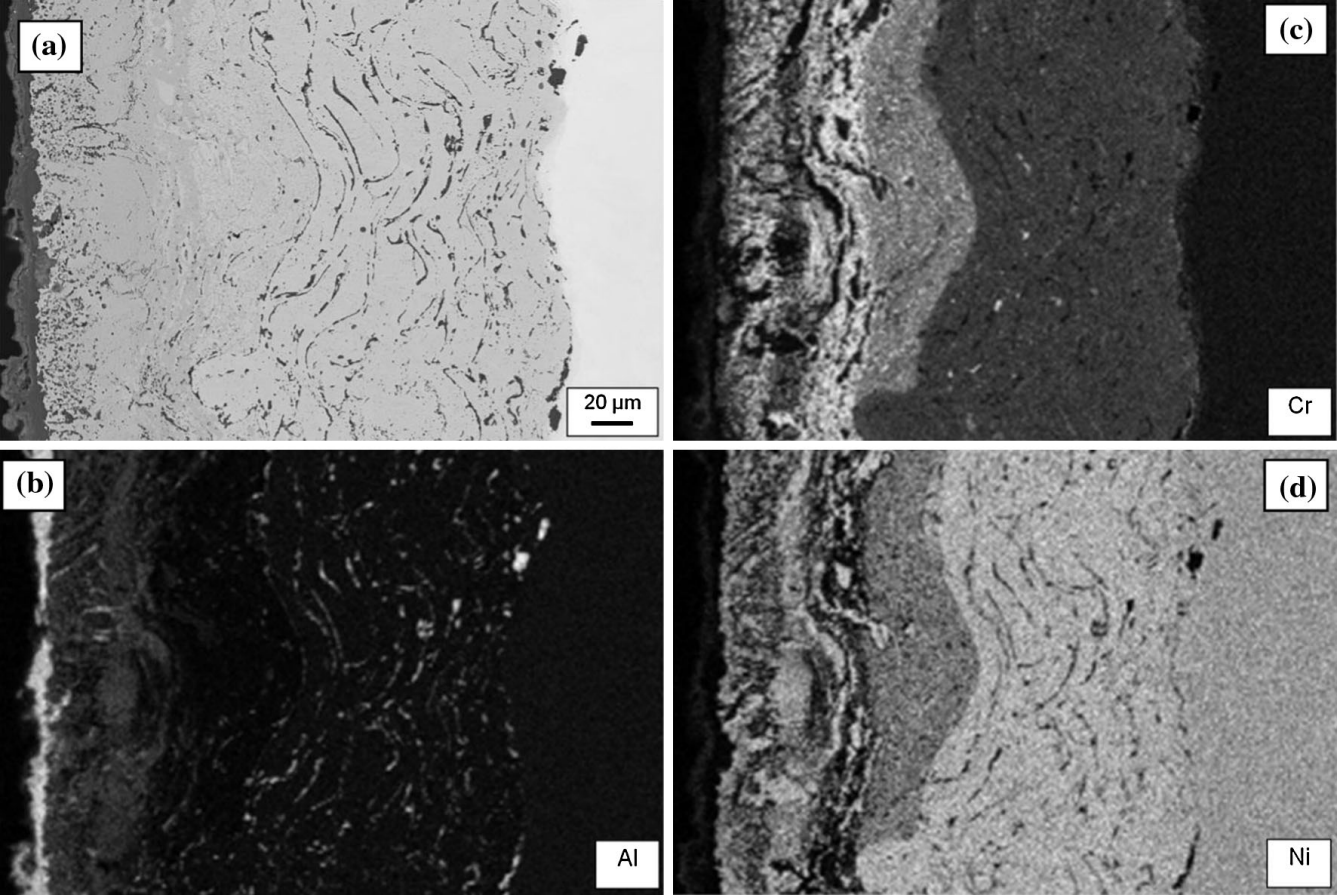
**a** Backscattered electron micrograph with associated energy-dispersive X-ray spectroscopy region of interest maps for **b** Al, **c** Cr and **d** Ni in Sample 1 which was aged at 700 °C for 500 h

Figure 4a shows a BSE micrograph taken from Sample 1 (700 °C/500 h) with corresponding EDS maps of Al, Cr and Ni shown in Fig. 4b–d, respectively. The chemical contrast in the maps reveals the existence of three layers of differing composition with significant variations in layer thickness which is consistent with the deposition technique applied in this case. The high-intensity layer seen at the left edge of the Al map (corresponding to a layer on top of the applied coating) is due to the presence of a thin oxide scale on the sample surface consisting mainly of alumina. Furthermore, alumina particles which have been trapped during the manufacturing process are discernible at the MCrAlY/substrate boundary, as well as within the body of the MCrAlY layer. The outermost (Al-rich) coating layer is clearly visible as a grey shade underneath the oxide scale in Fig. 4a. However, the Al levels remain fairly low in the rest of the structure. The Cr concentrations, however, show a significant presence in all layers, with the highest levels seen in a band of approximately 20 µm thick located towards the outer edge of the original high-Cr coating layer. Fairly high levels of Cr can be seen in the Al-rich layer, and its concentration declines through the MCrAlY thickness into the substrate. There is little evidence of the presence of Cr or Ni in the oxide scale layer, confirming that aluminium oxide is the dominant constituent of the scale. The Ni appears in the highest concentration in the MCrAlY layer, followed by the substrate and other layers.

**Figure 5:**
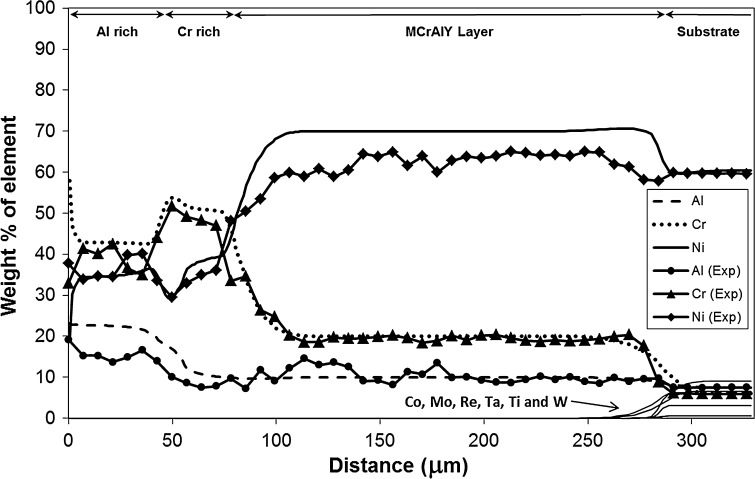
Experimental EDS matrix concentration profiles (Points) for Al, Cr and Ni, across the multilayer coating on Sample 1 (700 °C/500 h) overlaid with simulation profiles. The distance is measured from the original boundary between the oxide scale and outer layer

Figure 5 shows the concentration profiles of the key elements present in the coating structure, Al, Cr and Ni, obtained by EDS matrix profiling across the multilayer coating on Sample 1 (700 °C/500 h); the simulated profiles for the same elements are also superimposed. The averaging technique has reduced the scatter of concentration measurements which otherwise is present typically in EDS data obtained from overlay coating systems [29]. The EDS scans support the chemical contrast features shown in the EDS maps of Fig. 4b–d. The Al concentration profile supports the relatively high level of Al seen retained in the outer coating layer in Fig. 4b compared to the more homogenised levels in the rest of the coating/substrate system. The contrast variation in the Cr chemical map in Fig. 4c is also well explained by the EDS trace for Cr. A particularly interesting feature in the Cr profile is the slight accumulation of Cr in the Cr-rich layer near the boundary with the Al-enriched layer, which is coincident with a notable dip in the Ni concentration profile. In the map for Cr, in Fig. 4c, this corresponds to the ~20-µm-wide light-shaded band and the darker band in the Ni profile in Fig. 4d. The measured EDS profile for Ni corroborates the contrast features in Fig. 4d, with the general concentration increasing towards the MCrAlY layer.

The concentration profiles predicted by the model for the key elements Al, Cr and Ni agree remarkably well with the measured EDS traces. The ability of the coating structure to hold a high level of Al in the outer layer is indicated well by the model although the predicted concentration level is somewhat higher. The model output is also able to trace the Cr concentrations in the sample accurately, especially in the Cr-enriched and MCrAlY layers and the substrate. The concentration level and the location of the slight Cr-enrichment near the Cr-enriched layer boundary with the outer layer are predicted with a high degree of accuracy along with the dip in the Ni concentration at the same location. Despite the EDS profiles in the outer Al-rich layer showing more scatter, due possibly to the presence of oxidation products and the possibility of a complicated phase structure there, the concentration values predicted are in fairly good agreement with the measured mean values for this region. Although the measured Ni values are somewhat lower than predicted, the overall trend within the MCrAlY layer is faithfully reproduced by the model.

**Figure 6:**
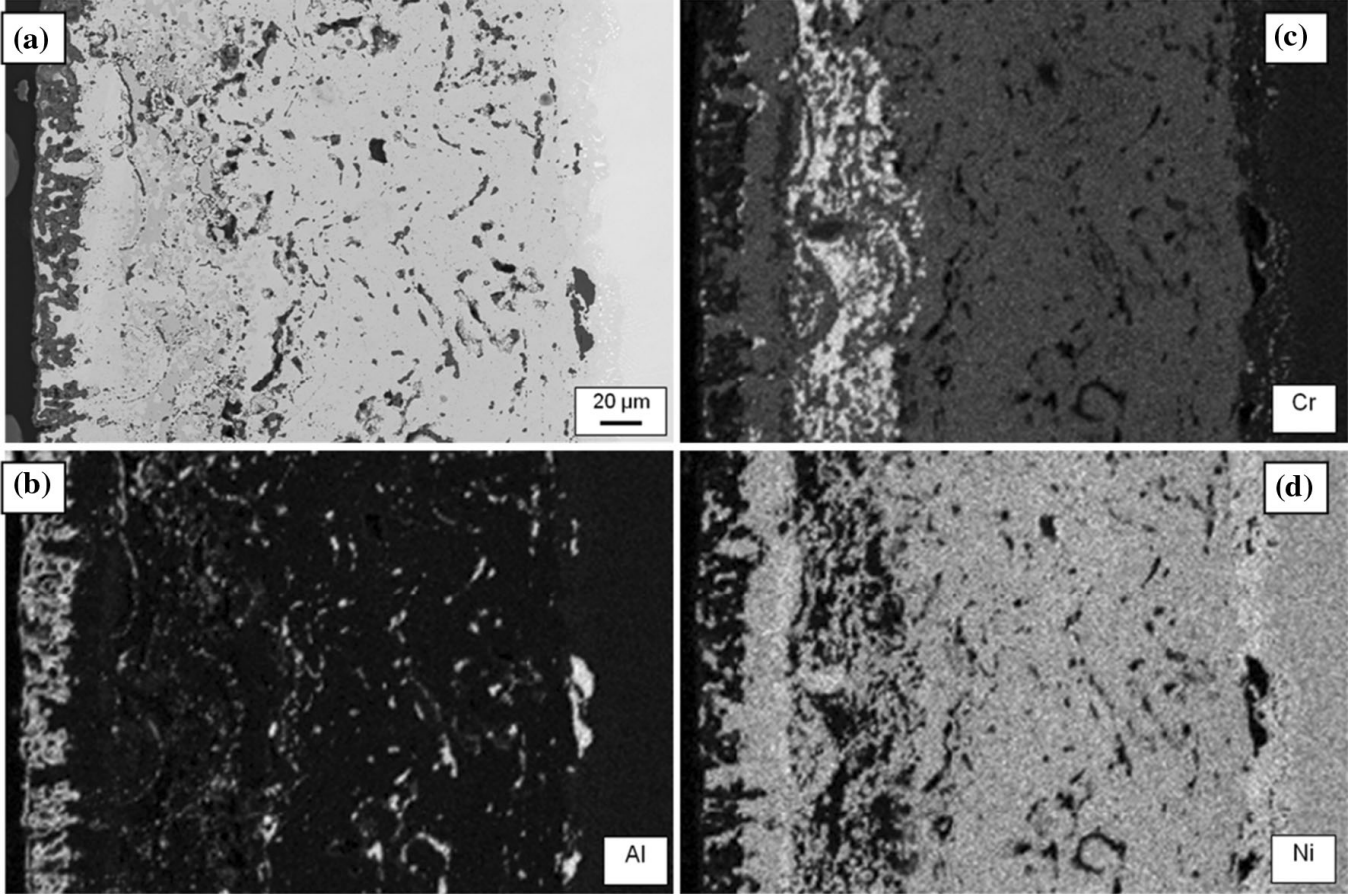
Backscattered electron micrograph with associated energy-dispersive X-ray spectroscopy region of interest maps of Sample 2 (900 °C/500 h)

Figure 6a shows a BSE micrograph and corresponding EDS maps, Fig. 6b–d of Al, Cr and Ni for Sample 2 (900 °C/500 h). The structure shows more uniformity in layer thicknesses compared to Sample 1 in Fig. 4, but there are other regions (not shown) which revealed uneven layer structures consistent with the manufacturing process. The elemental distribution in Sample 2 shares many features with Sample 1. In Fig. 6b, Al is heavily concentrated in the scale formed on the surface implying the formation of alumina, albeit considerably more compared to Sample 1 due to the exposure at a higher temperature. Entrapped alumina particles can be seen within the MCrAlY layer, and somewhat larger particles are concentrated at the substrate interface similar to Sample 1. Chromium distribution is concentrated in a tighter band within the Cr-rich layer in Sample 2, and Cr concentration is found to be much smaller in the Al-rich outer layer compared to Sample 1. Unlike in Sample 1, the maps of Sample 2 in Fig. 6 show that the alumina scale has penetrated considerably into the outer layer indicating that at 900 °C, internal oxidation is also occurring in addition to surface scale formation which was dominant at 700 °C.

**Figure 7:**
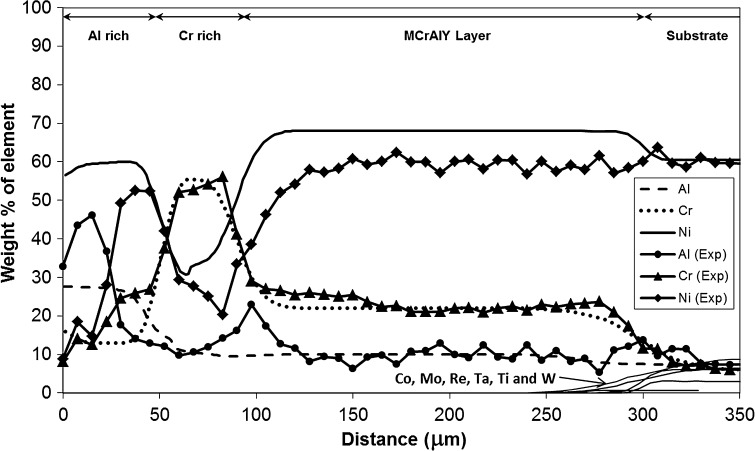
Experimental EDS matrix concentration profiles across the multilayer coating on Sample 2 (900 °C/500 h) are shown overlaid with simulation profiles. The distance is measured from the original boundary between the oxide scale and outer layer

Figure 7 illustrates the EDS concentration profiles of Al, Cr and Ni measured in Sample 2 (900 °C/500 h) along with the profiles predicted by the model. The EDS measurements show a sharp rise in Al concentration near the surface in the outer coating layer with an associated drop in Cr and Ni concentration levels. The high levels of Al, and lower Cr and Ni, are due to the fact that EDS data have been collected near an area where ingress of the alumina scale into the outer layer of the coating has taken place. The EDS profile reveals that a high amount of Cr still remains in the middle, Cr-enriched layer, although the peak concentration is somewhat lower than in Sample 1. In addition, the Cr amount retained in the outer Al-rich layer is much lower compared to Sample 1. There is a notable peak of Al at the Cr-rich layer boundary with the MCrAlY layer, which was absent in Sample 1. The Al level is flatter across the MCrAlY and substrate material as in the case with Sample 1.

The predicted profiles for Al, Cr and Ni in the MCrAlY- and Al-rich layers are in good agreement with the measured EDS scans, except in predicting the prominent small Al-peak at the MCrAlY/Cr-rich layer boundary. However, the predictions deviate considerably in the outer Al-enriched layer, as a result of the current model being unable to simulate the internal oxidation phenomena sufficiently which has occurred in this instance. Despite this deficiency, the model has predicted the chemical distribution fairly accurately at both 700 and 900 °C across the multi-layered system.

Neither the EDS profiles nor simulation predictions show the presence of an Al-depleted layer near the oxidation interface of the Al-rich outer layer which is often seen when an MCrAlY coating system is oxidised [11, 12]. The creation of an Al-depleted zone is dependent on two competing factors: (a) the depression of Al concentration at the external surface due to the removal of Al by the oxidation process and (b) the outward diffusion of Al through the coating layer as a result of the concentration gradient created by (a). Because of the extremely high concentration of Al present in the outer layer being examined, the lost Al is replaced fairly easily by diffusion and hence after 500 h no depletion zone is observed as a considerable amount of Al is still left in the coating layer.

#### Phase evolution

The phase structure of Sample 1 and 2 was analysed using a combination of SEM and TEM, and the observations have been compared against the predictions of the simulation model.

**Figure 8:**
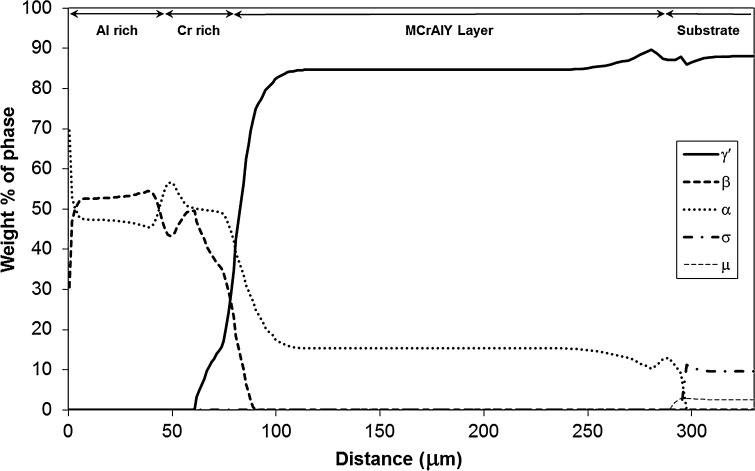
Phase profile predictions by the model for Sample 1 (700 °C/500 h). The distance is measured from the original boundary between the oxide scale and outer layer

**Figure 9:**
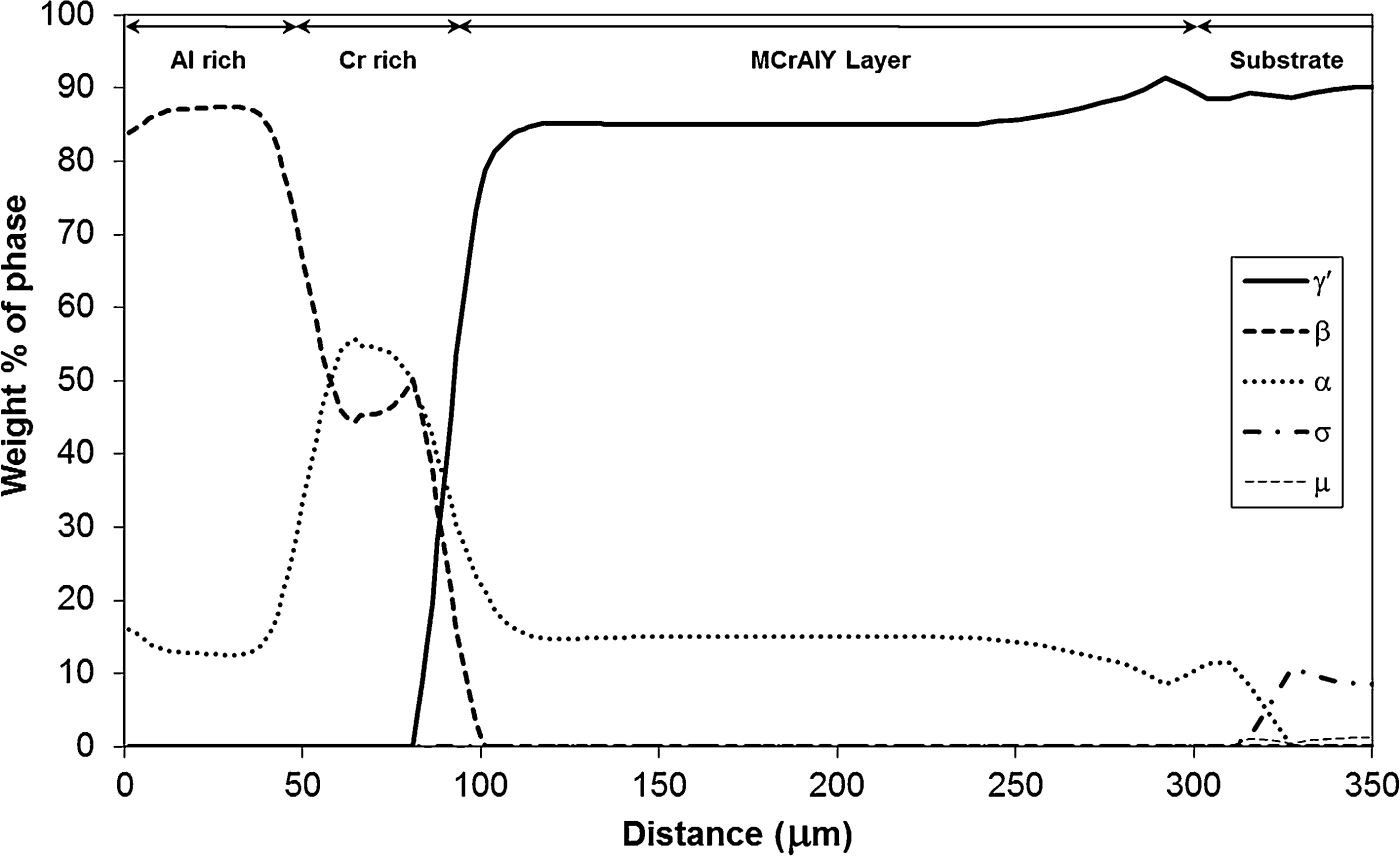
Phase profile predictions by the model for Sample 2 (900 °C/500 h). The distance is measured from the original boundary between the oxide scale and outer layer

Figures 8 and 9 show the phase profiles predicted by the simulations for Sample 1 and 2, respectively. In both samples, the coating structure mainly consists of the γ′, β and α-Cr phases, with small amounts of σ and µ TCP phases predicted in the substrate and substrate–MCrAlY interdiffusion zone. The α-Cr phase is present in all three layers of the coatings, and its concentration seems to follow the trend of Cr level of the sample (see Cr profiles in Figs. 5 and 7). The β phase concentration is the largest in the outer Al-rich layer and is also prevalent in the middle Cr-rich layer. The MCrAlY layer consists primarily of γ′ and also contains substantial amounts of the α-Cr phase according to the predictions.

#### Substrate–MCrAlY interface

**Figure 10:**
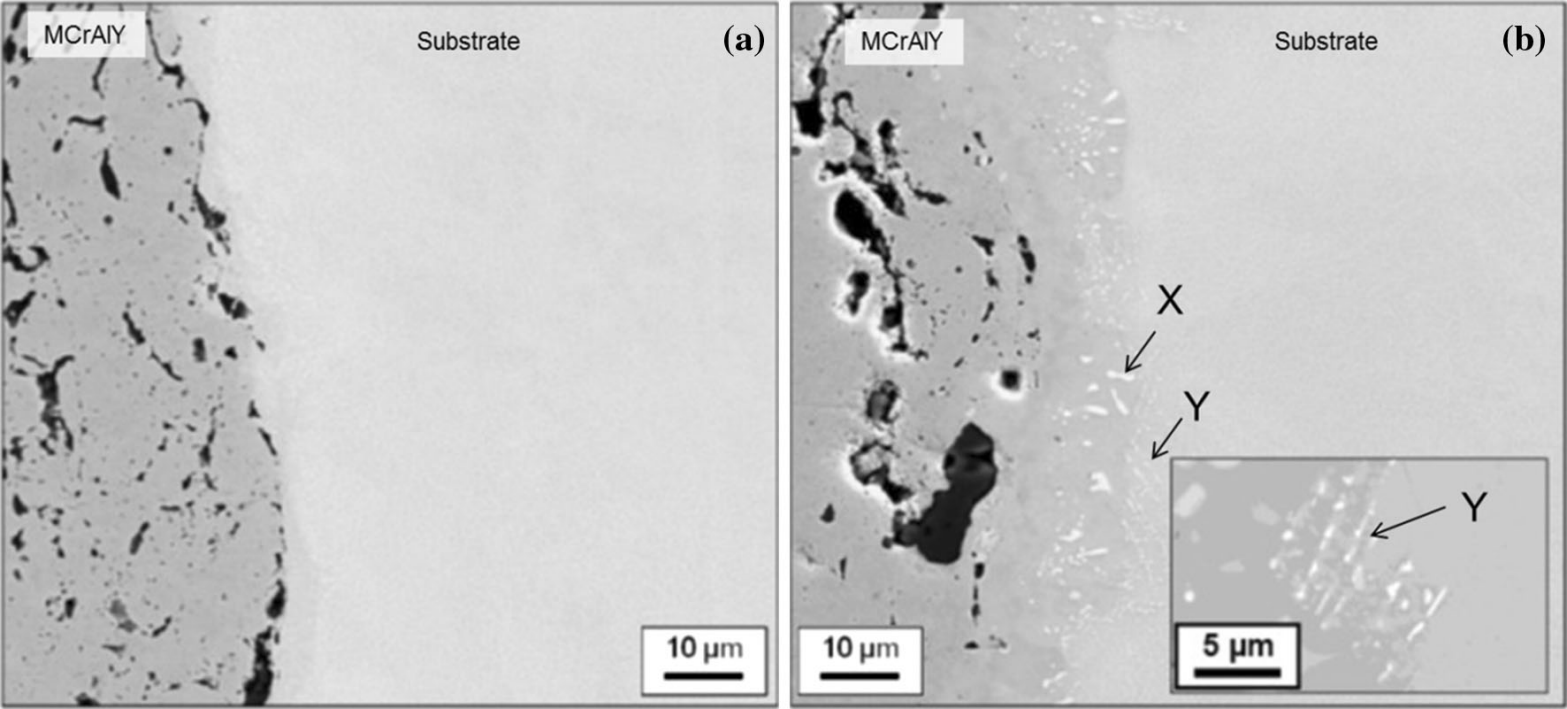
Backscattered electron image of the MCrAlY layer/substrate interface of **a** Sample 1 (700 °C/500 h) and **b** Sample 2 (900 °C/500 h) with higher magnification of substrate particles* inset*. Two types of bright particles identified as *X* and *Y* in (**b**)

Figure 10 shows BSE images obtained near the interface between the MCrAlY layer and the substrate. The dark features within the MCrAlY coating side of both samples were found to be either trapped alumina particles or voids which are characteristically present in overlay MCrAlY coating materials. Compared to Sample 1 (700 °C/500 h), Sample 2 (900 °C/500 h) which was treated at the higher temperature shows a clearly visible interdiffusion zone within which bright particles can be seen which are absent in Sample 1. There are two distinct morphologies for the particles in the BSE image—the first lies more towards the coating and is generally of a blocky elongated shape, measuring 1–5 µm in length and labelled as *X* in Fig. 10b. The second type is visible within the substrate near the interface region, labelled *Y* in Fig. 10b. These are much smaller and form strings of particles into the substrate, see inset in Fig. 10b.

**Figure 11:**
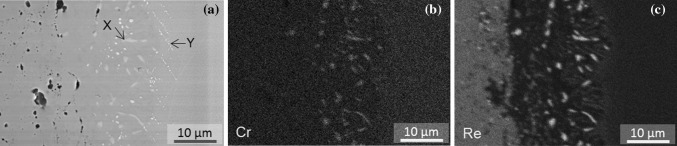
**a** Backscattered electron image with associated EDS region of interest maps of **b** Cr and **c** Re of the coating/substrate interface of Sample 2 (900 °C/500 h)

In Fig. 11a, a BSE image of this region is shown along with EDS maps for Cr and Re in Fig. 11b, c respectively. The large bright particles marked *X* in Fig. 11a reside mainly within the interdiffusion zone between the substrate and coating and are rich in both Cr and Re. Due to the relatively small size of these particles found on the substrate side of the interface, they cannot be detected clearly in the EDS maps shown here. Further EDS analysis with TEM of a number of these particles revealed them to be consistent with a cubic solid solution of Re in Cr having a Re content between 10 and 11 at.% (30 wt%), a small amount of Ni (1–4 at.%) and a balance of Cr. According to the Ni–Cr–Re–Al phase diagram [30], and the cubic nature of the phase, it is suggested that the phase is α-Cr (disordered BCC), with a relatively high amount of Re and a small amount of Ni in solution.

**Figure 12:**
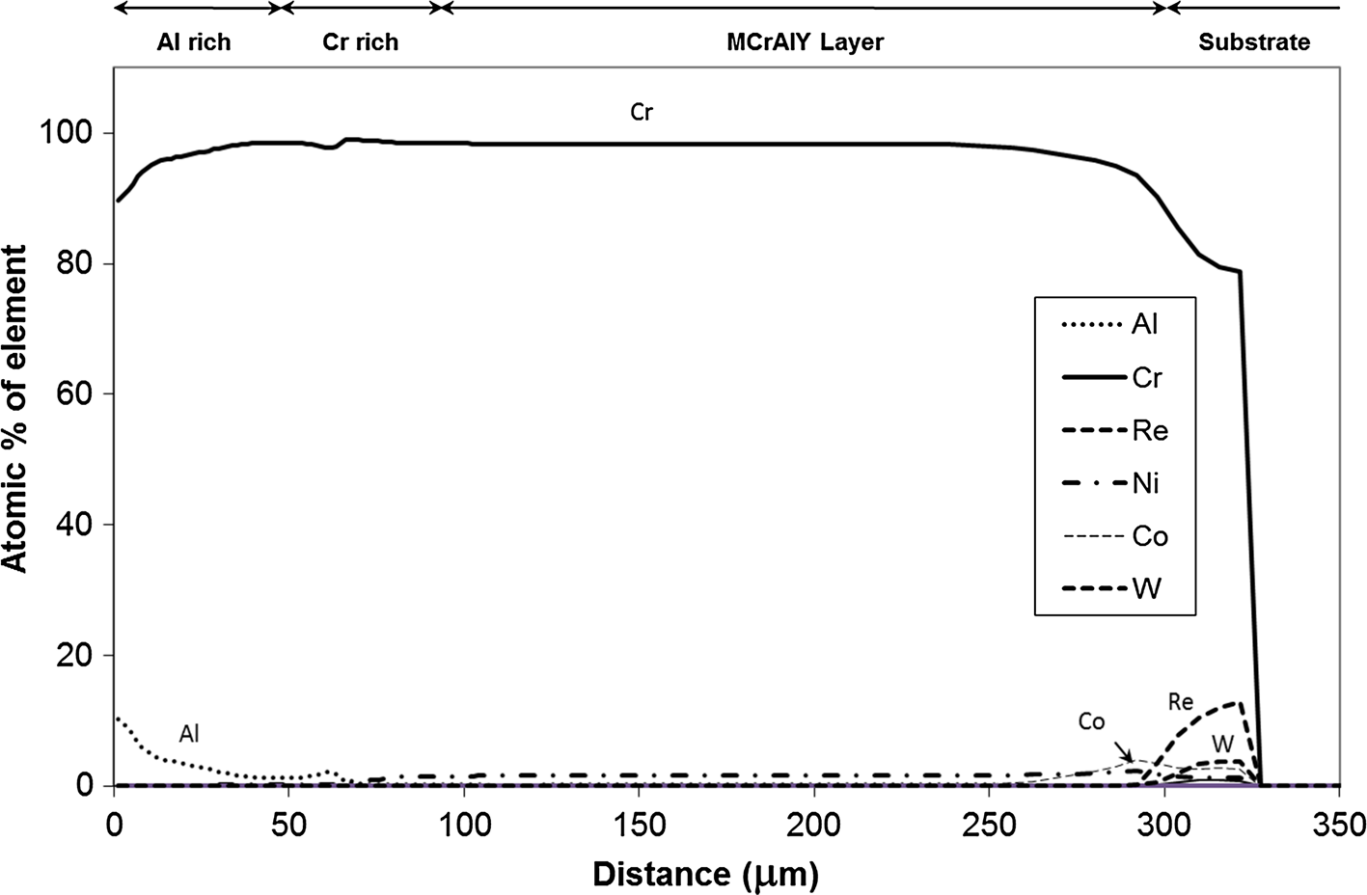
Elemental concentration variation in the α-Cr disordered BCC phase in the coating system and substrate in Sample 2 (900 °C/500 h). The distance is measured from the original boundary between the oxide scale and outer layer

Given the comparatively large amount of Cr in the MCrAlY coating, it is possible that a Re-rich α-Cr phase precipitates in time as a result of Re interdiffusion into the coating from the substrate. Examination of the predicted phase distribution for Sample 2 in Fig. 9 affirms the presence of the α-Cr phase extending well into the interdiffusion zone. Figure 12 shows how the simulation has predicted the chemical composition of the α-Cr phase to vary across the coating/substrate system for Sample 2 (900 °C/500 h). A closer examination of the constituent elements of this phase in Fig. 12 reveals that, in the interdiffusion zone, α-Cr phase dissolves a considerable amount of Re, in fact up to 12 at.%, which is well within the concentration range found by the EDS/TEM analysis.

**Figure 13:**
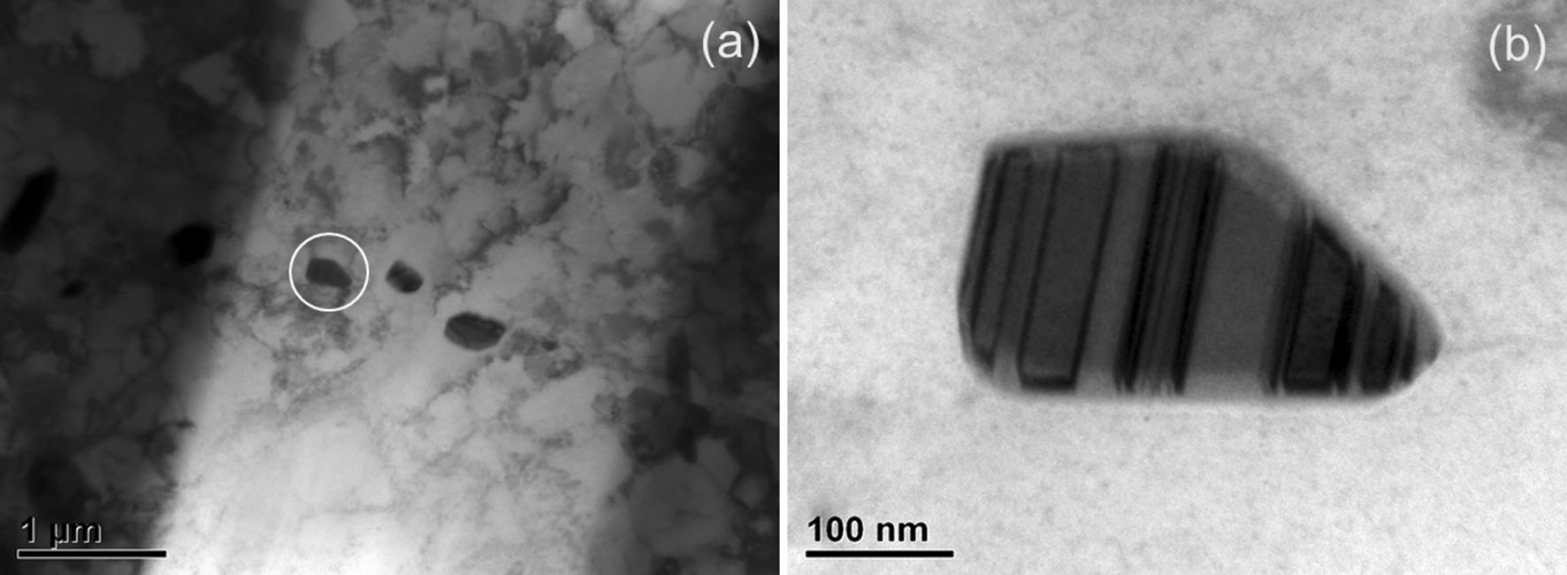
Bright-filed transmission electron micrograph of **a** three particles within the substrate closer to the MCrAlY layer of Sample 2 with higher magnification image of each of the particle indicated by the* circle* shown in (**b**)

On the other hand, the light-shaded particles (marked *Y*) seen on the substrate side of the MCrAlY/substrate interface in Fig. 10b seem to belong to a different phase. A bright-field TEM image of three such particles is shown in Fig. 13a, and a higher magnification image of one of the particles is shown in Fig. 13b. These particles typically measure 400–700 nm in length, and each has a highly twinned and striped appearance as evident in Fig. 13b. Such particles with this faulted appearance have previously been identified as the µ phase, e.g. [31]. The composition of each particle was measured using TEM/EDS, and the range of elemental concentration of them is given in Table 2. The chemistries of the particles are more complex consisting mainly of Cr, Re, Co, Ni and W, than those of the α-Cr particles which were Cr-, Re- and Ni-based.

**Table 2:** Chemical composition of the particles found near the substrate side of the MCrAlY/substrate interface compared with the composition of the candidate TCP phases (σ and µ) predicted by the simulation model for Sample 2 (900 **°**C/500 h)

	Element concentration (at.%)					Re + W
	Co	Cr	Ni	Re	W	
Measured	14.2–23.0	25.6–28.3	11.2–20.2	20.3–26.8	12.5–17.0	32.8–43.8
Predicted (µ)	15.3–18.3	22.8–29.7	13.3–14.8	8.7–12.0	26.4–27.8	35.1–39.8
Predicted (σ)	14.5–16.9	50.8–57.1	11.0–12.7	8.6–13.0	4.2–5.3	12.8–18.3

Indeed the predicted phase profiles of Sample 2 in Fig. 9 indicate the presence of both σ and µ phases, on the substrate side of the MCrAlY/substrate interdiffusion zone. The predicted composition of both of these phases varies from the interface towards the substrate, and the range of this variation is compared in Table 2 with the measured composition range of the particles. As can be seen, the range of the measured concentrations matches well with the predicted compositions of the µ phase except for the elements Re and W for which the simulation significantly over- and under-predicts the concentrations, respectively. However, it is well known that when present together, significant errors in individual concentration measurement of these two elements can occur due to EDS X-ray peak overlaps. Therefore, the sum of the measured concentrations for Re and W (final column of Table 2) has also been compared with the corresponding predicted values, and in this case, a good agreement can be found. The predicted composition of σ does not give as close a match as in the case of µ, in particular with respect to Cr and Re + W which are predicted in much higher and lower concentrations, respectively. Therefore, the composition match and twinned physical appearance suggest that the detected particles, *Y* in Fig. 10b, are µ phase.

#### Cr-rich zone

**Figure 14:**
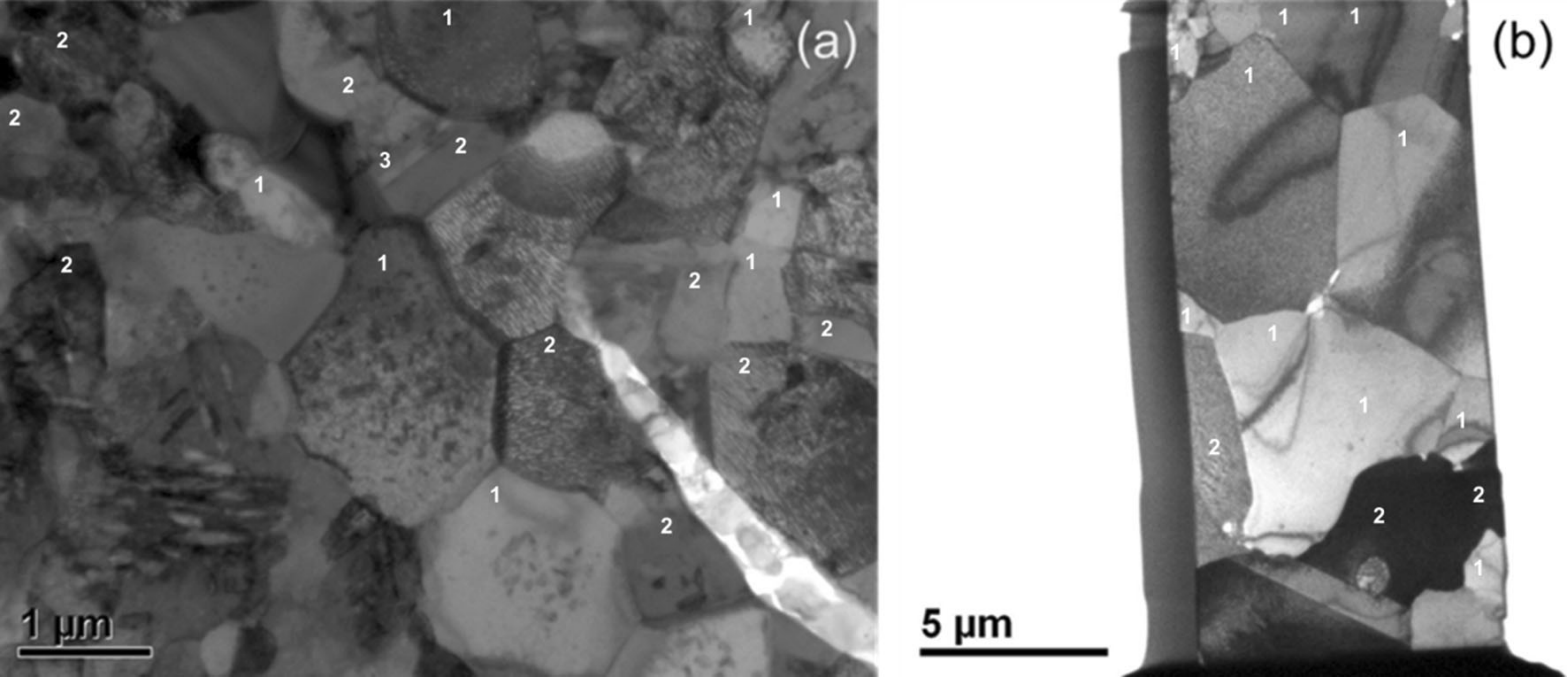
Bright-field transmission electron micrograph of the Cr-rich layer in **a** Sample 1 and **b** Sample 2

Figure 14a is a bright-field TEM image of a central part of the Cr-rich zone from Sample 1 (700 °C/500 h). From this region, three chemically discrete phases have been identified (numbered 1–3 in the image) with the compositions shown in Table 3. With the assistance of selected area diffraction, Phase 1 has been identified as α-Cr with small quantities of Ni in solid solution. As Fig. 8 shows, α-Cr is predicted in all three layers of the coating system and in the middle layer its percentage is at the highest. Therefore, the presence of this phase in the region is predicted accurately.

**Table 3:** Composition of phases found in the Cr-rich zone in Sample 1 (700 °C/500 h) and 2 (900 °C/500 h)

	Average concentration (at.%)			Number of grains	Concentration range (at.%)		
	Al	Cr	Ni		Al	Cr	Ni
Sample 1							
Phase 1	0.0	99.0	<1.0	17	0.0	97.0–100.0	0.0–3.4
Phase 2	0.5	34.0	65.0	19	0.0–6.34	30.0–36.0	62.0–70.0
Phase 3	0.0	63.0	37.0	3	0.0	59.0–64.0	35.0–41.0
Sample 2							
Phase 1	0.0	99.0	<1.0	10	0.0	93.3	100.0
Phase 2	20.0	10.0	71.0	3	19.1–20.1	8.6–11.4	70.7–71.7

The average concentrations, the number of grains of each phase analysed and their measured concentration range are given

Phase 2 contained Ni and Cr with a small amount of Al. Based on the Ni and Al concentration, it is possible that the phase is γ-Ni, despite it not being predicted by the simulations. However, the fairly strong 2:1 Ni to Cr atomic ratio suggests that it could also be the Ni_2_Cr phase which is known to form long range superstructures [32, 33] in some Ni- and Cr-containing alloys. However, the Ni_2_Cr phase has been reported to occur at much lower temperatures than 700 °C. The detailed crystallographic analysis required to clearly identify these precipitates is beyond the scope of this work.

Based on the observed stoichiometric ratios, Phase 3 can be identified as Cr_3_Ni_2_, which has been reported to occur in Ni-containing alloys with Cr [34]. Unfortunately, neither the Ni_2_Cr nor Cr_3_Ni_2_ phase is modelled in the thermodynamic parameter database used in the current simulations, and hence not predicted in the phase profiles in Figs. 8 and 9.

The bright-field TEM micrograph for the Cr-rich zone of Sample 2 is shown in Fig. 14b. Here, there is evidence of considerable grain growth relative to Sample 1, with an equiaxed grain size of up to 5 µm compared to approximately 1 µm in Sample 1. Within this region, two different phases can be distinguished, as labelled in Fig. 14b and their measured compositions are listed in Table 3. The high amount of Cr in Phase 1 suggests it to be α-Cr, and Phase 2 has a composition consistent with the γ′ phase. Both these phases are predicted for the Cr-rich layer by the simulation model according to the profiles shown in Fig. 9.

#### Al-rich layer

**Figure 15:**
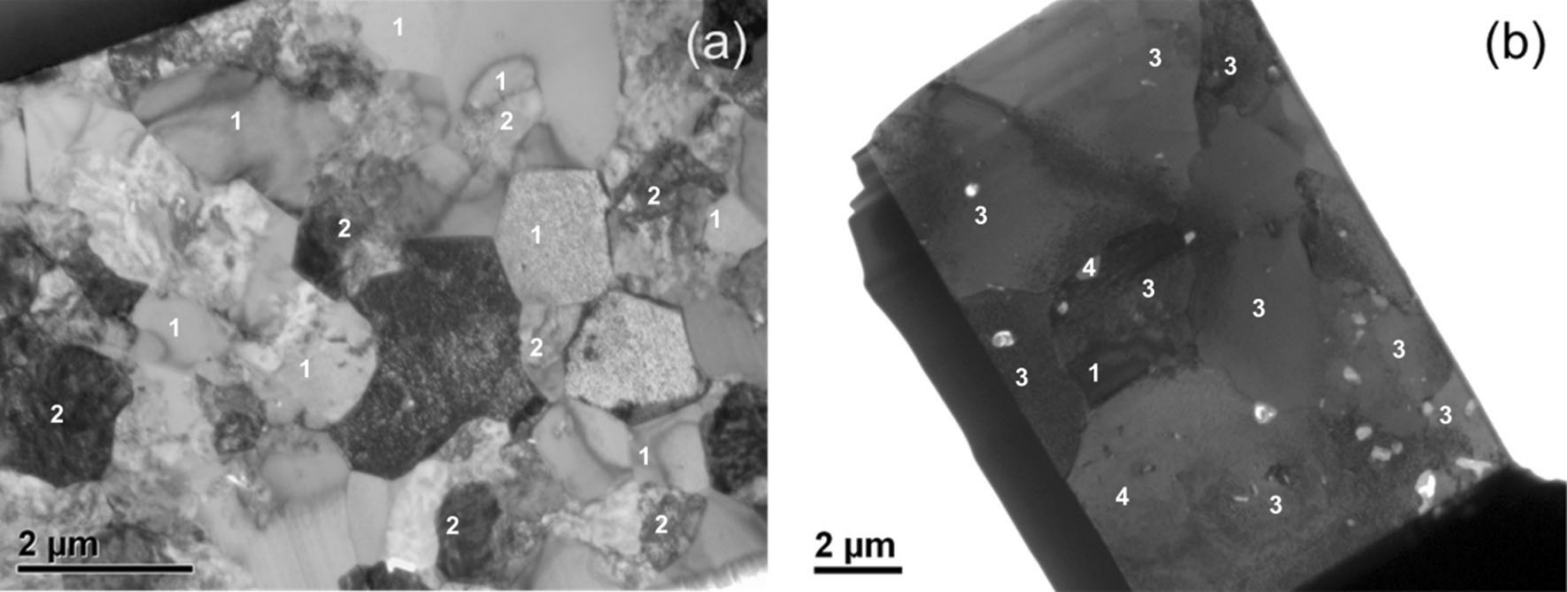
Bright-field transmission electron micrograph of the Al-rich layer in **a** Sample 1 and **b** Sample 2

A bright-field TEM image of the Al-rich layer of Sample 1 is shown in Fig. 15a. The sample consists of two different types of grains as identified by the labels. EDS analysis revealed that the majority of the grains were β-NiAl (label 1) while others (label 2) were a mixture of β and α-Cr phases. These phases are predicted accurately for this region by simulations in phase profiles depicted in Fig. 8.

In Sample 2 (900 °C/500 h), the grain structure is considerably coarsened compared to Sample 1, as shown in Fig. 15b. Chemical analysis of the Al-rich layer of Sample 2 revealed the presence of three phases which are identified by labels as (1) β, (3) Ni_2_Cr and (4) γ′. The simulation output shown in Fig. 9 predicts only β and α-Cr phases for the sample. It could be possible, however, that since Ni_2_Cr phase is not included in the thermodynamic database, the phase structure seen here is not accurately forecast by the model.

The amount of Al lost from the outer layer in the scale-forming process can be easily determined using Eq. (2). Assuming a density of 3.75 g/cm^3^ for the scale, the model gives an Al loss of 0.09 and 0.41 mg/cm^2^ at 700 and 900 °C, respectively.

## Summary and conclusions

A coupled model which was originally developed for predicting microstructural evolution in bond coats and superalloy substrates has been extended to simulate an experimental multilayer coating system on a nickel-based superalloy. The model combines diffusion and surface oxidation kinetics with equilibrium thermodynamics to predict the development of concentration and phase profiles using the ageing history of the multilayer system. The coating structure studied consisted of an Al-rich outer layer, Cr-rich inner layer and MCrAlY layer on top of a Ni-based superalloy. The two samples studied had undergone a controlled ageing at 700 and 900 °C for 500 h which was simulated by the model.

The model-predicted concentration profiles for each ageing condition were compared with EDS measurements, and good agreement was found for the key elements of the multilayer structure, Al, Cr and Ni. The phase evolution predicted by the simulations was compared with the microstructure revealed at key locations of the samples using SEM and TEM techniques. Good agreement between modelling and experimental results was found for a number of important phases.

**Figure 16:**
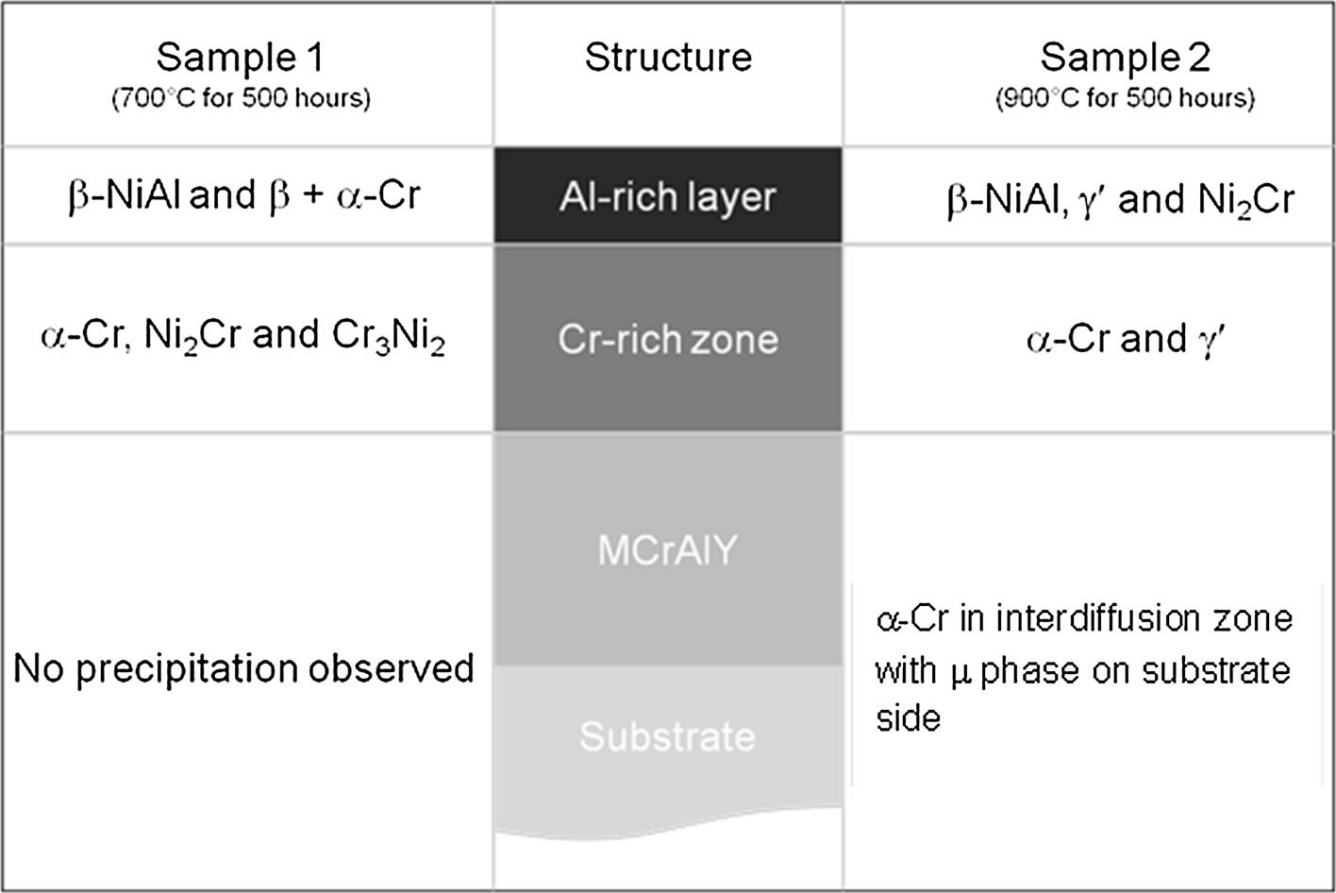
Schematic of the coating structure with the identified phases present within Samples 1 and 2

The experimentally observed phase composition of the coating system is summarised in Fig. 16. The outer Al-rich layer of Sample 1 is composed primarily of β-NiAl with some grains consisting of a mixture of β and α-Cr. The Al-rich layer of Sample 2 is comprised of three phases identified as β-NiAl, γ′ and Ni_2_Cr. The Cr-rich middle zone of either sample has a structure mainly of α-Cr. The Cr-rich zone in Sample 1 has some Ni_2_Cr and Cr_3_Ni_2_ phases whereas Sample 2 has γ′. At the substrate/coating interface, Sample 1 shows no significant precipitation whereas two distinct types of precipitates have been observed in Sample 2. These have been identified as a solid solution of α-Cr with dissolved Re on the coating side of the interface, and the µ phase on the substrate side.

The ability of the simulation model to predict many microstructural features accurately in the system studied here shows the potential of the current model to be a valuable design tool for further development of multi-layered coating systems.
